# Caregivers’ reading practices to promote literacy in a South African children’s home: Experiences and perceptions

**DOI:** 10.4102/sajcd.v65i1.559

**Published:** 2018-06-28

**Authors:** Faadhilah Tayob, Sharon Moonsamy

**Affiliations:** 1 Department of Speech Pathology and Audiology, Thelle Mogoerane Regional Hospital, South Africa; 2 Department of Speech Pathology and Audiology, University of the Witwatersrand, South Africa

## Abstract

**Background:**

Exposure to social trauma influences the psychosocial experiences of vulnerable children. This affects their positive development, consequentially resulting in poor scholastic progress. South Africa’s history of inequality and injustice has compounded the current social, educational and economic situation, highlighting the need for research on children in care. A paucity of published studies exists on caregiver facilitation of literacy skills among vulnerable populations in South African children’s homes. The purpose of this paper is to describe the reading practices that caregivers in one children’s home (orphanage) used to promote literacy development.

**Method:**

An exploratory, descriptive contextual design was implemented, using inductive and interpretative approaches. Semi-structured interviews and focus group discussion were conducted. Ten caregivers, who supervised children aged 9–10 years at the home, consented to be participants. Applied content thematic analysis was used to interpret the data obtained.

**Findings:**

The caregivers at the children’s home were implementing some reading strategies, but they did not engage sufficiently in self-reflection on the reading processes. The caregivers used relevant reading strategies, such as asking questions to develop understanding and memory recall. They encouraged dialogue through characterisation, where the children acted out the roles of the main characters. These reading strategies demonstrate the quality of the mediation.

**Conclusions:**

Speech–language therapists have a role in prevention and promotion programmes in children’s homes. They should advocate for, collaborate on and support caregivers’ facilitation of early literacy skills in these homes, as the link between literacy and language cannot be ignored. Providing guidelines and sharing knowledge on reading instruction for the children are essential in improving the literacy rates in vulnerable populations. Language and literacy interventions are only effective and meaningful if the social and cultural contexts are considered. Such interventions would add value and constitute a step towards redressing past inequalities in South Africa. These results contribute to our understanding of context when developing literacy programmes. The sample size was a limitation. However, the aim was not about generalisation but to gain an insight into caregiver reading practices so that literacy programmes are built on these strengths.

## Contextual background: Vulnerable children in South Africa

Some South African families, specifically mothers and their children, infected and/or affected by HIV and/or AIDS, are provided with safe living environments. Several of these children have been orphaned and live in care after the death of their parents (UNICEF, 2012). An estimated 3.7 million orphans live in South Africa, with only a percentage of them residing in children homes, while others may be living with relatives or running child-headed households (UNICEF, 2012). The plight of orphans and abandoned children is an ever-increasing phenomenon, globally. A study conducted by Morantz and Heymann (2010) revealed that orphaned children experience a sense of isolation that limits effective coping and grief recovery. The psychosocial experiences of these vulnerable children influence their educational and future achievements.

Vulnerable children need safe environments, but given South Africa’s past, it is often not the case. From its apartheid past, South Africa has inherited a legacy of inequality and social displacement, resulting in high levels of poverty, violence, neglect and substance as well as sexual abuse (Graven, 2013; UNICEF, 2012). Theron and Theron (2014) argue that children growing up in such contexts experience social trauma that affects their positive development and consequentially results in poor scholastic progress. International declarations, including the Millennium Goals and Education for All, aim to prioritise education of vulnerable children (Escueta, Whetten, Ostermann, & O’Donnell, 2014).

South Africa’s history of inequality and injustice has compounded the current social, educational and economic situations, highlighting the need for research on children in care. Studies have been conducted internationally and locally on topics related to education or literacy particularly, as well as on the contexts of children’s homes (orphanages) (Escueta et al., 2014). Nevertheless, a paucity of published research exists on caregiver facilitation of literacy skills among vulnerable populations in South African children’s homes. We argue that creating an enabling environment with quality mediation will improve the culture of literacy in children’s homes.

Therefore, the purpose of this paper is to examine the reading practices of caregivers that promote literacy in a children’s home. Findings from the study will contribute to promoting collaborations between speech–language therapists (SLTs) and caregivers, as well as designing language and literacy interventions relevant to the context. The word ‘children’s home’, as used in this paper, is synonymous with the global term ‘orphanage’. The theoretical framework and key concepts that underpin this paper are discussed, next.

### Reading acquisition and development

The importance of both reading and writing is well documented in the literature; however, for the purpose of this paper, an argument is made for reading acquisition and development. Schools provide formal reading instruction, but the home environment needs to support reading development, so that reading skills are consolidated (Centre for Education, 2017; Mather, Bos, & Babur, 2001). Additionally, home environments provide the source that enhances language skills, which are foundational for reading acquisition (Sènèchal, LeFevre, Thomas, & Daley, 1998). Parents and caregivers from different social classes have different perspectives concerning the importance of literacy development and facilitation, as literacy is socially bound (McKenzie, 2015). High levels of illiteracy among a large section of the South African population are mainly because of the country’s historical inequalities and social injustices (Graven, 2013). Hence, huge disparities in knowledge around reading exist. These views strongly influence parental and caregiver roles in facilitating quality mediation in literacy development. Thus, some parents and caregivers might be unable to facilitate effective reading within their homes. Collaborative support by professionals can only be provided if the current reading practices in children’s homes are identified.

### Reading strategies that promote literacy development

Studies on reading have examined different methods of interaction between caregivers and children that influence emergent and existing literacy skills. These methods of communication between caregivers and children include verbal interaction (Vernon-Feagaans & Bratsch-Hines, 2013), utilising environmental print (Neumann, 2014), exposure to writing (Welsch, Sullivan, & Justice, 2003) and using gestures during early word learning (Bennet, Weigel, & Martin, 2002). Specific reading strategies, such as shared book reading, dialogic reading and print referencing, also facilitate and encourage literacy development (Graves, 2006; Owens, 2014). Furthermore, environmental print can foster print awareness, print knowledge and sound knowledge. Neumann (2014) indicates that environmental print is one avenue to overcome a lack of resources and has had effective results, especially in younger children. Environmental print may be beneficial in the context of children’s homes, as it is functional and cost-effective and can be implemented by all caregivers during routine activities of play, bath and meal times.

The shared book reading strategy fosters social and affective aspects of literacy, which expands the child’s understanding of language structures such as narratives, vocabulary, syntax, story structure and basic print concepts (Owens, 2014). Shared book reading allows children to form a connection between oral languages and print (Roth, 2009). Dialogic reading is a form of shared book reading, which is known to be a one-on-one interactive picture book reading approach. Graves (2006) states that the dialogic reading strategy promotes language development in children through the practices of using language to talk about language (metalinguistic skills), providing feedback on language and scaffolding interactions between the adult and the child. Print referencing is another strategy that highlights verbal and non-verbal cues used by adults to focus children’s attention on important aspects of the text. Children are encouraged to track print and pointing to the print and pictures. Lane and Wright (2010) state that print referencing aims to increase the metalinguistic focus of reading aloud and as a result increases print interest. The use of print referencing further develops children’s knowledge of print concepts, concept of letters and words and alphabet knowledge. Finally, decoding strategies and discussion of the text will benefit emerging and novel readers, improving their comprehension of the text. Mature readers develop a knowledge base of narrative and expository text structure, which aids their future reading comprehension (Gajria, Jitendra, Sood, & Sacks, 2007; Harris & Rothstein, 2014). When parents and caregivers have some awareness of such strategies, they can promote reading. However, this is not always evident, as certain factors facilitate reading acquisition and development, while others may limit its trajectory. Thus, parent/caregiver support might be interrupted as a result of certain proximal factors.

### Factors affecting reading acquisition and development

Various proximal factors, including the home environment, parent education, social and economic disparities, affect a child’s reading acquisition (Brittnacher, 2014). According to Neumann (2014), language and literacy skills of children from socially disadvantaged communities (SDC) are lacking, as they may have less opportunity to access quality mediation and resources. Poor facilitation of language and literacy development will influence children’s learning and subsequently their academic performance (McDowell, Lonigan, & Goldstein, 2007). Academic failure begins early and might persist throughout a child’s schooling career. We argue that the need to promote quality caregiver–child interactions is foundational for language and literacy development. A longitudinal study by Juel (1988, cited in Colvin, 2007) found that children who lack decoding and comprehension skills at the end of Grade 1 are likely to lack these skills as they progress to higher grades, falling behind their peers. Lyon et al. (2001, cited in Colvin, 2007) state that reading difficulties may persist into adulthood, unless suitable intervention is provided. Therefore, early and appropriate learning opportunities ought to be endorsed, in addition to the quality of mediation from significant adults or peers. Mediational theory that underpins the interaction between adult and child is discussed next.

### Mediation for reading

To understand a mediated experience, Vygotsky’s theory on the zone of proximal development (ZPD) (Kozulin, 2015), as well as Feuerstein’s mediated learning experience (MLE) (Tzuriel, 2013; Tzuriel & Caspi, 2017), are introduced. MLE is defined as the quality of the interaction between an individual and their environment (Feuerstein, Feuerstein, Falik, & Rand, 2006). MLE is a dynamic and intentional process conducted by a more knowledgeable other within the ZPD. Literacy development can therefore be facilitated through MLE, as caregivers interact with the children. Vygotsky and Feuerstein’s theories, as indicated in research (Scanlon, Anderson, & Sweeney, 2011), support the argument that the quality of the learning experiences that children are exposed to is the main contributing factor to effective learning and scholastic progress. Reese and Cox’s study (1999) confirmed that the quality of adult book reading in the home environment had an effect on children’s literacy skills. Children’s responses to parents’ and caregivers’ interactions depend on the quality of these experiences, hence the focus on the caregivers’ reading practices within the orphanage. Thus, by examining the reading practices of the caregivers, an insight into the quality of the mediation will be achieved. However, the quality of mediation needs to be established with further research.

### Home and social contexts

The interaction of parents and caregivers with their children is frequently dependent, as indicated earlier, on proximal factors such as parent educational levels, employment status and social circumstances. These proximal factors may marginalise children in SDC, placing them at higher risk for reduced language and literacy acquisition. Limited exposure to literary environments (e.g. books, comics, written puzzles and games) is a significant contributor to low literacy levels (Graven, 2013). A preschooler with fewer opportunities for acquiring knowledge and skills, and limited access to books and reading, is more susceptible to presenting with reading difficulties than a preschooler from a rich literacy environment (Snow, Burns, & Griffin, 1998). All children, regardless of their socio-economic background, ought to benefit from rich literacy environments, as it encourages their language and literacy skill development and ultimately influences their scholastic achievements. It is the quality and not just the quantity of stimulation or their socio-economic background that makes a difference to their literacy development (Bennet et al., 2002; McDowell et al., 2007).

We maintain that children, despite their adversities, are resilient and will achieve success given the support, opportunities and the environment to discover learning, particularly in literacy. Hence, the caregivers in children’s homes play a fundamental role in creating an environment that stimulates literacy development. Their practices in encouraging reading need to be explored, so that strengths are acknowledged and the interventions requested are relevant. As the home environment is the context in which literacy is first encountered (Bennet et al., 2002; Niklas & Schneider, 2013), examining the children’s home context, especially in South Africa, is important. Furthermore, it is fundamental to establish what caregivers do to promote rich language and literacy development and to determine how they consider the children’s diverse strengths and weaknesses when learning.

The information obtained through addressing the study’s aims will foster an asset-based approach, so that literacy programmes that enhance current caregiver practices are the focus. Identifying issues of facilitation and resources are critical considerations when discussing literacy acquisition and development in the context of children’s homes, as interruptions in stimulation may occur frequently when these children are placed in foster care and/or returned to their relatives. Caregivers have indicated a need for up-skilling with regard to promoting language and literacy among the children. This will redress the issues of social injustice and financial constraints experienced in children’s homes. Various low-cost solutions to resources can be applied, but the critical skills of mediation (that is, the quality of the interaction) and reading strategies that emphasise how to read more effectively are foundational. Caregiver understanding of the importance of literacy plays a role in what they actually do to facilitate and promote literacy.

Therefore, the aim of the study examined the caregivers’ reading practices that promoted literacy in a children’s home in Gauteng. The following objectives operationalised the main aim:

- to determine the caregivers’ perceptions of reading as a strategy to promote literacy
- to establish how reading was encouraged by the caregivers
- to determine the reading strategies caregivers use during shared book-reading experiences
- to establish the resources available in the children’s home that promote literacy.

## Method

### Research design

An exploratory, descriptive contextual design was implemented, using a quantitative and qualitative approach. Focus groups and semi-structured interviews provided triangulation of methods to ensure validation of data collected and to capture a more complete and contextual representation of the study.

### Participants

A purposive, non-probability sampling strategy was applied. The sample consisted of 10 caregivers in the age range of 20–50 years old, who supervised children between 0 and 9 years of age. They were selected according to the inclusionary and exclusionary criterion. The participants had to be resident at the selected children’s home and had to interact daily with the children they supervised. Either one or two caregivers supervised each age group. In the case of two caregivers, both the caregivers were included, if they agreed to participate. Furthermore, the caregivers were not excluded based on their level of English linguistic competence, as interpretation services were available when needed.

### Procedure

An additional caregiver who had met the inclusion criteria was interviewed for the pilot study but was not included in the main study. Feedback from the pilot study included modifying and clarifying questions. For the main study, each participant was interviewed individually and thereafter one caregiver supervising each age group was randomly selected to join the focus group. Face-to-face semi-structured interviews of approximately 15–20 min were conducted at the children’s home. The interview schedule examined the participants’ demographic information, their knowledge of reading strategies, reading resources and how the caregivers facilitated reading. The questionnaire further explored the caregivers’ thoughts on reading in English, versus other indigenous languages, types of text structures and the influence reading had on caregivers’ knowledge and skills.

An interpreter was included in one of the 10 interviews conducted, once consent was obtained from the interviewee. A 45-min focus-group discussion was also conducted at the home, 2 weeks after the individual interviews. The focus group schedule was based on the themes obtained from the interview data.

### Data analyses

The data was analysed using content thematic analyses. Themes were formulated from the data. Three phases were included in the thematic analysis: coding, which aims to organise the data; categorising, to distinguish between the various ideas of the data collected; and identification of themes (Creswell, 2009).

### Trustworthiness and credibility

Credibility was established through a pilot study, as well as triangulation of methods. Face-to-face interviews and formulating a focus group, reduced intrinsic bias, which results from single method studies. The participants reviewed their interview transcripts (member checking) to ensure clarity of the data collected. Honesty of response was encouraged to establish creditability. Transferability and dependability were achieved by providing detailed information on the methodology and the background of the study, as well as through audio recording.

## Ethical consideration

All ethical parameters were maintained. The participants provided informed consent and the institute’s committee on human research approved the study’s protocol (protocol #H15/02/32).

## Findings

The study examined the caregivers’ reading practices that promote literacy in a children’s home in Gauteng. The findings are presented and discussed according to the themes that emerged, in line with the objectives.

### Caregivers’ perception of reading

The majority of the caregivers felt that reading was important, as it allowed the children to improve their English language skills, increased their knowledge base on different topics and promoted their education and future job opportunities.

‘I think that because in class they are being taught in English, so they understand better what the teacher is explaining in the class by reading and they get to learn more.’ [P9, female, caregiver]

The caregivers’ perception of English as a strategic avenue to educational success was evident. From their explanations, reading was linked to the enhancement of English as a language, rather than being perceived as an avenue to developing the children’s phoneme–grapheme awareness, encoding, decoding and print awareness. Some of the caregivers, however, highlighted the importance of reading in relation to literacy development, as indicated in P8’s and P6’s statements:

‘I think it’s [reading] very important because it helps you, really mostly in speaking; you can speak very well if you read (mostly, eh) those novels or magazines. It helps you to know things that are happening outside and the spelling; also if you like to read, I don’t think you can have a problem with spelling.’ [P8, female, caregiver]

‘It will help them because if you are able to read words, word by word, your spelling will be good because you went through the words while we were reading and you know the word while we’re doing the spelling.’ [P6, female, caregiver]

These caregivers saw a relationship between speaking, reading and spelling, as well as building world knowledge.

Additionally, oral narration and reading of text structure were indicated as important. The caregivers suggested oral narration as important, consolidating the oral culture of Africa. They indicated that old traditional tales were not often found in books, as these are stories told over generations that impart life lessons.

‘Yes, because you get more of a vocabulary and you learn a lot of things when you are reading, like the feelings and actions of people. Like what happened before – the history.’ [P7, female, caregiver]

These findings indicate that both reading and oral narration are important for literary knowledge, even though their structures are different (Dickinson, n.d.). The caregivers described books that portrayed historical facts and mentioned oral stories as a medium for transferring cultural practices; both modes seemed to engage the different generations in discussions.

The importance of text structure when reading was nevertheless not ignored among the caregivers. They reported that text structure enables children to spell words and to understand grammar.

‘They read it so nice and loudly. They take the book and sometimes call a friend, “did you know this and this?”, and you can see he is really trying to share the information that he/she is picking up from the book.’ [P2, female, caregiver]

Unbeknown to the caregivers, they were exposing the children to different narrative and expository text structures, which would influence the children’s reading comprehension, as indicated in the studies by Gajria et al. (2007) and Harris and Rothstein (2014).

### Reading empowers caregivers

The caregivers described reading as benefitting their roles as caregivers. They stated that reading developed their interpersonal communication skills. They were better able to interact with others, especially those with whom they worked. They also experienced increased confidence, where they were able to transmit their knowledge when responding to the children’s questions:

‘For me, before I came here I never used to read much, but with English, it improves the language and helps you understand better. I mean, we help the children with their homework, and how are we going to help them if we don’t read? So reading is helping me get better in English for myself and as a caregiver.’ [P5, female, caregiver]

The caregivers said that they could apply the knowledge gained from reading to scenarios that they may experience within the home context. Additionally, they learnt how to nurture and care for the children, understanding their behaviour.

‘As an individual it helps me with my English, you know; it helps me understand not only myself but also how to interact with other people that I work with and my teamwork in general. As a caseworker it helps me because most of the time with the kids it is not only the information I learned before but sometimes you really need to read and go back to the materials that you had so that you can understand what to apply when those scenarios happen.’ [P2, female, caregiver]

#### Caregivers’ encouragement of children’s reading

Caregivers emphasised the importance of reading to the children. They practised various methods, including bedtime stories, setting up a mini-library within each cottage where books were available and within reach, and setting up a reading time. Caregivers felt that reading should be encouraged through the methods that most motivated the children. In the focus group, they stated that having pictures together with text improved the child’s comprehension of the text.

‘Yes, putting up written words with a picture to explain so that the child understands. Remember, if it’s a picture, it is more attractive to the child than just having straight words. So putting a picture and a few sentences under to explain the picture I think will also encourage them, especially the younger ages.’ [P2, female, caregiver]

The caregivers indicated that pictures were attractive and engaging, which motivated the children to read. The children discussed the pictures in shared book reading, aiding in their understanding of the text.

Moreover, the older children were encouraged to assist with the younger children during reading time. Hence, they read occasionally to the younger children, and this often inspired the younger children to want to read.

‘There is normally paired reading. So normally, when they ask the careworkers and it is too much workload then they usually take the older kids to help them to read to the younger kids.’ [P2, female, caregiver]

‘When the elders are helping them with spelling, they respond. They like to be taught to read today and then the following day they want to read themselves; they don’t want anyone to help them.’ [P10, female, caregiver]

The caregivers believed that the younger children enjoyed learning from the older children; consequentially they attempted to read independently. Young children also learnt related literacy skills from older children, including spelling strategies. However, the caregivers recognised that younger children respond positively or negatively, depending on whether they like or dislike the person reading to them.

The caregivers said that setting an example of their own reading behaviours was important. Eight caregivers mentioned that the type of reading materials being read by the adults and caregivers around the home influenced the children’s perceptions of the importance of reading, while two caregivers indicated that the reading materials they read did not influence the children.

‘Well, what I can say is that if they saw me reading a magazine or something, they are only interested in the pictures not the information.’ [P1, female, caregiver]

‘I think that whatever you are reading is important because you obviously want to know what it is about and you want to have information. And you want to understand the book that you are reading, the stories and everything. (If they perceive reading as important it will always be important to them.)’ [P6, female, caregiver]

During the focus group discussion, the caregivers confirmed that they often encountered learning experiences when reading and interacting with the children. They indicated that it was important to share these learning experiences with the children through praise and acknowledgment, as this was motivational for them.

‘I just praise them and say “Wow I didn’t know it is like that” and then they will say “really but you are so old” and then I will say “yes I am old but I didn’t know” and then they will be surprised because they have taught me something that I didn’t know and I have informed them that I learnt from them. They will feel proud of themselves.’ [P8, female, caregiver]

These comments indicate that the caregivers understood the importance of acknowledging and connecting with the children, which builds a sense of belonging for vulnerable children.

The findings revealed that the caregivers had access to a variety of reading materials, and the children were often aware that their caregivers enjoyed reading. The children residing at the home allowed the caregivers space and time to read, and they were often curious and enquired about what the caregivers were reading. The caregivers’ reading behaviours confirmed that leading by example is an important teaching tool; they were seen demonstrating the reading behaviours that they were promoting. Moreover, the caregivers’ encouragement reflects the humanity they demonstrated through the process of reading, thus creating a nurturing and mediated environment.

#### Caregivers’ beliefs on the frequency of reading

**FIGURE 1:**
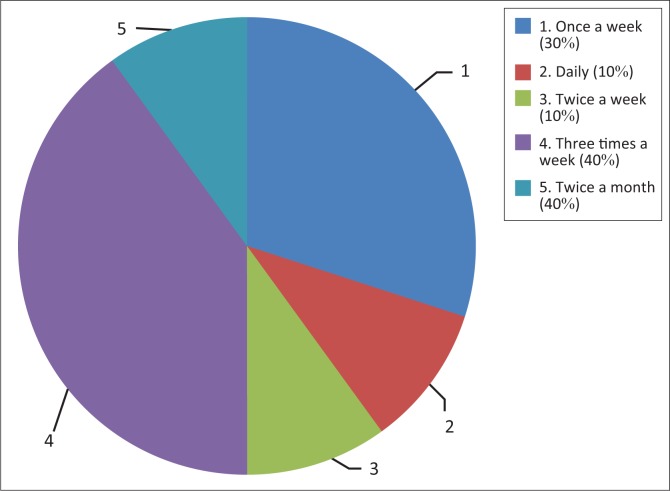
Frequency of reading to the children.

The results showed that all caregivers read to children during the evening, mostly before the children went to bed. However, reading did not occur on a daily basis and varied among caregivers. The following findings, recorded in Figure 1, indicate the frequency of reading times within the caregivers’ respective cottages.

Figure 1 indicates that a greater number of caregivers (40%) found reading to the children three times a week to be beneficial, whereas one caregiver believed that reading should occur more often. According to Li, Zhang, Chin and Khalid bin Bari (2013), an increased frequency of reading activities leads to positive reading outcomes. The focus-group interview confirmed that reading to children three times a week was sufficient, as the children engaged in additional activities during the remaining time. The caregivers suggested that in addition to reading, children should socialise with others, play, share their feelings, role-play and colour pictures. This, they stated, ought to keep the children motivated to read. The caregivers stated that children do learn skills of understanding other individuals’ feelings and perceptions and sharing when engaging in the activities mentioned; hence they promote a balance between reading times and additional activities.

#### Reading strategies applied by caregivers

Four caregivers acknowledged the term ‘reading strategies’, while approximately 60% (*n* = 6/10) had never heard the term before. One caregiver stated that she was unsure of whether or not she had heard the term ‘reading strategies’ before. The four caregivers who indicated their awareness of reading strategies described it as a method of teaching a child how to read based on their age, identification of the type of books being read and a method of learning. They said that reading strategies enabled the child to give meaning to and explain what was being read. The caregivers, within the focus group, further explained that they were aware of reading strategies such as picture reading, group reading, storytelling and the use of age-appropriate books. The six caregivers who had no previous recall of the term stated that reading strategies might be defined as the way in which one reads, reading in groups and using a dictionary to identify and understand unfamiliar words and explain what is being read. These responses indicate that the caregivers have some understanding that strategies are techniques to aid the child’s reading. However, their own level of metalinguistic skills and their English linguistic proficiency might have limited their explanations.

The most common reading strategies that the caregivers reported on were dialogic reading and shared book reading. From the discussion, it was found that the caregivers used the terms (dialogic reading and shared book reading) as per the interview questions and they presented their understanding of these strategies based on a literal interpretation of the name of the strategy. Therefore, dialogic reading was often described as the use of dialogue imitation while reading; and shared booked reading was described as the sharing of books among one another. This confirms that the caregivers did not have an understanding of these specific strategies. However, their engagement with the children during book reading and discussion reflected their shared book reading strategies.

**FIGURE 2:**
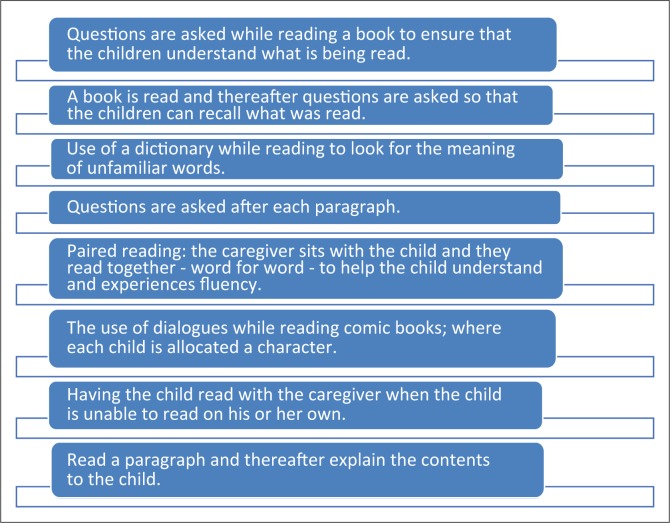
Reading strategies implemented by the caregivers.

A number of additional reported strategies implemented by the caregivers are listed in Figure 2.

It was concluded that the best strategy they applied with the children who they supervised was print referencing, which involves reinforcing the child by encouraging them to continue reading and picture reading. Implementation of reading strategies, especially referring to pictures, was deemed important by the caregivers, as this strategy enabled the child to understand the text being read:

‘It is more interesting for the kids if the book has pictures so that they can see what the story is talking about’ [P1, female, caregiver].

P1 reported that the child becomes familiar with the image, then formulates and maintains a mental image while reading. Furthermore, the child has a wider view of what is being read when pictures are used in comparison to text only. This discussion of reading strategies confirms the caregivers’ positive perceptions of the reading.

When these strategies are examined, it is clearly evident that even though the caregivers might not have the language competency to define strategies used, they are applying many useful cues to aid reading. They used questions and explanations to build comprehension of the text. They created opportunities to recall the text being read, which develops memory and expressive skills. Further, they encouraged the children to use a dictionary to build their understanding of words. They might also not have been aware that the act of reading together promotes fluency in reading, nor that interest and motivation are built when assigning the child a choice of character.

The findings also showed that the caregivers thought that using reading strategies promoted self-confidence in reading and increased interaction with others. They stated that children became aware of dialogue, learnt from what they read and were encouraged to ask questions. Increased self-confidence encourages children to read by themselves, promoting a love for reading. Furthermore, individual reading assists in literacy development, as it allows the child to understand that they can gain knowledge through reading and can read for pleasure. Engagement in individual and group reading sessions varied among caregivers.

### Reading materials and literacy resources

**FIGURE 3:**
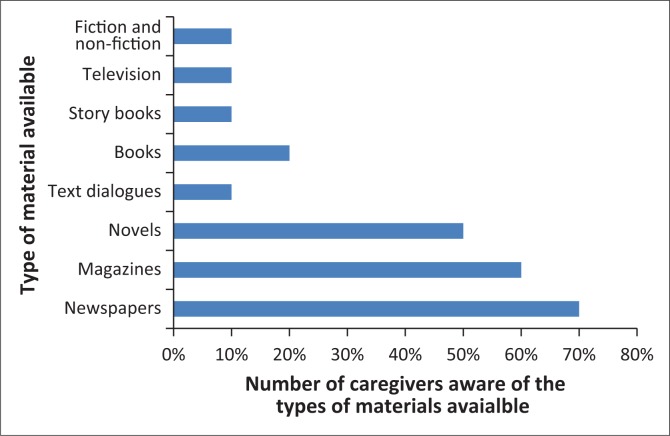
Caregivers’ awareness of the types of literacy resources.

The participants reported that they had access to a variety of reading materials available in the library at the home. Figure 3 indicates the types of materials available at the children’s home.

The majority of the materials at the home are only available in English. Two caregivers reported that there were some materials in isiZulu, while another caregiver reported that Sesotho, isiXhosa and Afrikaans materials were available, though limited. Hence, the caregivers did not read to the children in languages other than English.

The caregivers felt that reading materials in their indigenous languages ought to be available to the children, as many of them had difficulty with understanding English. The caregivers stated that many of the children residing in the home were from Zulu and Sotho backgrounds, and English was not their mother tongue. Materials in their home languages would thus promote the preservation of indigenous languages and cultural practices, in addition to English.

One caregiver said:

‘… it is important for these children to have access to their home languages, as it builds on their cultural knowledge, as the children may one day return to their communities.’ [P2, female, caregiver]

Another caregiver noted:

‘Yes, it’s good, because remember, our kids are – it’s not like they are here forever; definitely one day they have to go back to the society and the society they come from is not an English-speaking society; it can be a Zulu or a Sotho, or isiXhosa [*sic*].’ [P4, female, caregiver]

Furthermore, it was found that isiZulu was one of the languages being taught at school. The caregivers said that the children only had access to isiZulu word lists but not to reading materials, resulting in limitations of learning and applying the language adequately. However, one caregiver felt that having the reading materials in English only was beneficial; she believed that English was a priority as it was the language of learning and teaching (LoLT) at school. Moreover, she said that English would be beneficial for the children’s future, in tertiary education and job opportunities. The majority of the caregivers expressed the importance of materials in both the children’s mother tongue and English, reflecting equal value of all languages. The single caregiver who indicated only English reading materials might be unaware of mother tongue attrition and its impact on cognitive and linguistic skills. Hence, collaboration between caregivers and SLTs is important, where SLTs can share their understanding of first and additional languages in language and literacy development (Jordaan, 2015).

## Discussion and conclusion

The reading practices of the caregivers provided an insight into how they promoted reading in this children’s home. This offers a lens into formulating appropriate collaborative support structures for the SLT and the caregivers. The caregivers’ reading practices were evident and did promote literacy development across the age groups. Reading interventions built on a strength-based approach will empower caregivers and enhance the literacy skills of the children.

The quality of the caregivers’ mediation is foundational to the success of literacy development in children’s homes, as these children are vulnerable and require nurturing environments (Feuerstein et al., 2006; Morantz & Heymann, 2010). The findings in this study confirmed that the caregivers had created an enabling environment, and this had promoted literacy development for their resident children. The caregivers’ perceptions of reading reflected fundamental phases that would create stable and consistent stimulation, encouraging reading. Important distinctions, such as oral narration versus text reading, showed that the caregivers were responsive to their context. This was further indicated when the caregivers discussed the need for access to more resources in the children’s mother tongue, as languages and cultures have to be retained, even though English was the children’s LoLT at school. Such insights are assets to encouraging reading skills as part of social development (Dickinson, n.d.; McKenzie, 2015).

Caregivers are role models, setting an example by scheduling reading times for both children and adults. It was our perception that the caregivers expressed great personal gains from reading, which fostered their confidence, translating into information sharing with the children. Moreover, the children in this home seemed to be secure in asking questions related to the readings, indicating an environment of ease around reading time. Creation of a library facility within the home environment further cemented the importance and value of reading. The caregivers’ responses showed that they had created an environment that supported literacy development. Li et al. (2013) supports the call for an enabling home literacy environment and encourages parents and teachers in Singapore to have this realisation. In addition, a responsive environment that provides structure and a positive climate facilitates language and literacy development (Roberts, Jurgens, & Burchinal, 2005). These facilitating behaviours of the caregivers in the current study represent a quality interaction, which is foundational for language, literacy and scholastic success. Scanlon et al. (2011) support the argument that the quality of interaction between adult and child influences scholastic success.

The caregivers used relevant reading strategies, such as asking questions to develop understanding and memory recall. They encouraged dialogue through characterisation, where the children acted out the roles of the main characters. These reading strategies achieved the aim of developing literacy skills through quality mediation. The main aim was thus met, as the study described the reading practices that caregivers applied in the children’s home to facilitate reading instruction.

The findings also revealed that the caregivers implemented specific reading strategies, even though they seemed unaware that they were in fact doing so. Despite the facilitation of these reading strategies being devoted for the most part to improving the understanding of the English language, caregivers were meeting the needs of the children, regarding their learning and wanting to read. The caregivers’ absent knowledge of text labels, including print awareness, print knowledge, sound awareness, phoneme–grapheme awareness and syntax, might be construed as a poor knowledge base and thus reduced mediation, but we disagree. It is therefore important to identify strengths in a context prior to formulating intervention plans.

Thus, we concluded that SLTs have a role in prevention and promotion programmes in children’s homes. They can work in collaboration with the caregivers, to further literacy practices. SLTs need to apply an asset-based approach to build on the potential of the caregivers. SLTs have a knowledge base of the relationships between language, cognition and literacy, and the theories of learning. Moreover, they have a solid background in promoting and maintaining the importance of the mother tongue in young children, as the first language scaffolds the development of additional languages (Jordaan, 2015). The caregivers had a wealth of knowledge on the context, the resources and the children. Thus, through collaboration, sharing and developing skills, indicate dual benefits. SLTs would empower caregivers’ facilitation of literacy skills, influencing the children’s scholastic performance. Moreover, SLTs would develop their own skills in formulating relevant programmes and being responsive to the context (Moonsamy & Kathard, 2015). Language and literacy intervention will only be effective and meaningful if the social and cultural milieus are considered. Such interventions would add value and would constitute a step towards redressing past inequalities in South Africa.

A language barrier was a limitation in one interview, but an interpreter was included to manage the interaction. The results of the study may not be generalised, as they relate to one context. Nevertheless, the findings contribute to understanding the context of children’s homes and caregiver reading practices that promote literacy. Future studies may look at caregiver and parent perceptions on reading strategies, following literacy interventions.
